# Large Scale Bacterial Colony Screening of Diversified FRET Biosensors

**DOI:** 10.1371/journal.pone.0119860

**Published:** 2015-06-10

**Authors:** Julia Litzlbauer, Martina Schifferer, David Ng, Arne Fabritius, Thomas Thestrup, Oliver Griesbeck

**Affiliations:** Max-Planck-Institut für Neurobiologie, Am Klopferspitz 18, Martinsried, Germany; CNR, ITALY

## Abstract

Biosensors based on Förster Resonance Energy Transfer (FRET) between fluorescent protein mutants have started to revolutionize physiology and biochemistry. However, many types of FRET biosensors show relatively small FRET changes, making measurements with these probes challenging when used under sub-optimal experimental conditions. Thus, a major effort in the field currently lies in designing new optimization strategies for these types of sensors. Here we describe procedures for optimizing FRET changes by large scale screening of mutant biosensor libraries in bacterial colonies. We describe optimization of biosensor expression, permeabilization of bacteria, software tools for analysis, and screening conditions. The procedures reported here may help in improving FRET changes in multiple suitable classes of biosensors.

## Introduction

FRET (Förster Resonance Energy Transfer) between mutants of the Green Fluorescent Protein GFP and its variants has become a preferred mode to monitor protein interactions and conformational change in many different applications [1,2]. A growing number of genetically encoded biosensors have been created that exploit FRET as ratiometric read-out mode [3,4]. Sensors created cover a multitude of biological signaling events, ranging from calcium indicators [5,6,7] over reporters of cyclic nucleotide levels [8,9] and metabolites [4] to indicators for kinase activation [10,11,12], as examples. Changes in FRET within these sensors rely on alterations of the distance and the orientation of the donor and acceptor fluorescent protein to each other. Targeted protein engineering has been used to increase these FRET changes by altering linker sequences between fluorophores and ligand binding domains, mutating hinge residues within domains, or incorporating combinations of fluorescent proteins with various circular permutations, thus providing a range of angles and orientations between fluorophores to choose from. Optimizing such changes has been time consuming and labor intensive. Recently, engineering of FRET biosensors has incorporated methods of library generation and screening to optimize response properties. Libraries consisted of combinations of circularly permutated donor and acceptor fluorophores [13,14,15,16,17] and/or choices of extended, flexible or rigid linkers [18,19,20,21], typically comprising not more than a few hundred mutant sensors.

Screening larger numbers of sensor variants is, however, very desirable. Molecular biology conveniently allows generating large numbers of sensors diversified on the DNA sequence level. For example, randomization of only a short stretch of 4 amino acids already result in a library of 20^4^ variants. The challenge subsequently lies in identifying sensors with large FRET changes in such libraries where linker sequences between the fluorophores and the ligand binding domain have been diversified. This presumably results in a large variability of angles and orientations of donor and acceptor fluorophores to each other, both in the ligand free and ligand bound state. Therefore techniques are necessary that allow recording fluorescence of large numbers of sensor variants both in the ligand free (the minimal ratio Rmin) and ligand bound (the maximal ratio Rmax) state and retrieving the sensors that show the largest FRET change after binding of ligand. Bacterial colony screening is a cost-effective means to screen large numbers of biosensor variants in an acceptable time. *E*. *coli* strains can be transformed at high rates, and each bacterial colony expresses but a single sensor variant. On a single agar plate of 10 cm diameter, up to one thousand bacterial colonies each expressing a different sensor variant can be grown side by side, distinguished and imaged using suitable wide-field optics. Moreover, DNA coding for interesting sensors can be easily isolated from bacteria for further analysis, be it sequence analysis, recombinant protein purification for in vitro analysis or subcloning for expression in mammalian cells. By targeting libraries of single fluorophore calcium sensors to the periplasmic space of *E*.*coli*, Zhao and colleagues [22] could use bacterial colony screening to survey non-FRET biosensor variants in both ligand bound and ligand free states. As FRET sensors so far cannot be targeted efficiently to the periplasmic space, other strategies are required to optimize screening protocols for this class of biosensors. Here we present procedures for large scale screening of FRET biosensors by mutagenesis and wide-field bacterial colony screening. This protocol can be adopted for the optimization of FRET changes in several types of FRET biosensors.

## Methods

### Molecular biology

To generate a sensor library for subsequential screening two randomized linkers were integrated into the corresponding prototype sensor constructs. These linkers flank the ligand binding domain N- and C- terminal, which is sandwiched between the fluorescent proteins. In order to randomize both linkers simultaneously, while not committing to “fixed” amino acids by incorporating restriction sites, the seamless ligation strategy described by [23] was utilized. Specifically two components were combined in a SLiCE reaction: the linearized vector backbone, which incorporated both fluorescent proteins at its N- and C- terminus respectively and the ligand binding domain, which was flanked by the two linker regions. Starting with a prototype construct which was cloned via BamHI and EcoRI into pRSETB (Invitrogen), the vector backbone was linearized via PCR with outwards facing primers, amplifying from the N- terminus of the second fluorescent protein (e.g. mCitrine) to the C- terminus of the first fluorescent protein (e.g. mCerulean3). In parallel the ligand binding domain was amplified with primers that consisted of 3 distinct parts. The first part being a 20 base pair region at the 3’ end that is reverse complementary to the respective 5’ ends of the ligand binding domain, to facilitate amplification. The second part follows in 5’ direction and incorporated the randomized amino acids for the linker region. Between 1 and 4 degenerated codons of the type NNB (where N codes for either A/T/G/C, B codes for C/G/T) (or VNN for the respective reverse primer with V coding for G/C/A) were used. The NNB codon can encode all 20 amino acids while reducing the frequency of stop codons. The last region at the 5’ end consisting of 21 base pairs was homologous to the ends of the linearized vector backbone and promotes seamless ligation in the SLiCE reaction, which was performed according to the protocol provided by Zhang et al. [23]. Subsequently 1 μL of the reaction mixture was transformed into chemical competent *E*.*coli*, which were then plated on selective agar medium.

### Bacterial plate screening

Libraries were transformed into *E*.*coli* XL1-Blue cells or *E*.*coli* BL21-Gold cells (both Stratagene) and plated on LB agar plates containing ampicillin (100 μg/ml) with a desired colony density of ~700–800 colonies per plate. They were incubated at 37°C for 16–18 hours, and subsequently stored at 4°C overnight for further maturation. Before imaging, colonies were blotted onto filter paper (Whatman 3MM) pre-soaked in MOPS buffer (30 mM MOPS, 100 mM KCl, pH 7.5). For the experiments on aptamer induction colonies were left on agar plates as the procedure lasted for several hours.

Bacterial colonies blotted on filter paper were mounted on a maneuverable stage and imaged with a CoolSNAP ES2 CCD camera (Photometrics). The excitation and emission filter wheels, and shutters were controlled by a Lambda 10–2 optical filter changer (Sutter Instrument). A Lambda LS/30 Stand-Alone Xenon Arc Lamp (Sutter Instrument) was used as a light source, which transmitted the light with a liquid light guide. The excitation filters used were D436/40x (CFP excitation) and HQ500/20x (YFP excitation), in combination with the emission filters D480/40m (CFP emission) and HQ535/30m (YFP emission). The imaging system was controlled by a custom program written in Python. Saturation of calcium sensors was achieved by permeabilizing the cells with a solution containing poly-L-lysine and ionomycin (50 μg/ml each, in MOPS buffer) for five minutes and subsequent application of a calcium solution (100 mM, in MOPS buffer). Expression of the ten copies of the RNA aptamer which could bind to the aptamer binding peptide Rsg1.2 was accomplished by treating the cells with anhydrotetracycline (20ng/μL). Solutions were initially applied by spraying five times with 30 ml polyethylene spray bottles (Rotert, Germany), which had a volume per spray of 0.15 +/- 0.05 ml. After improving administration of solutions to bacterial colonies we were taking aim for the center of the blotting paper with an attached laser pointer and sprayed solution once, for 2 seconds, with a spray gun (JetStream I, K350, HVLP).

Data analysis was carried out by a custom written program in Python, which identified the 1–2% best performing colonies on a plate and their position for subsequent picking. Alternatively, R_0_ of each sensor was plotted against its ΔR/R in Origin 8.1., and new sensor variants were compared to their parental sensors.

### Expression and purification of proteins and *in vitro* measurements

For protein expression, the plasmid encoding the sensor was transformed into BL21-Gold cells, which were grown in an autoinductive LB medium supplemented with ampicillin (for autoinduction: 0.6% glycerol (v/v), 0.2% lactose (w/v), 0.05% glucose (w/v), 50μg/ml ampicillin, based on (Studier 2005)) at room temperature for ~ 60 hours. Cells were pelleted by centrifugation (5500 x g) and subsequently resuspended in resuspension buffer (20 mM Na_2_HPO_4_, 30 mM NaCl, 20 mM imidazole) supplemented with protease inhibitors (1 mM PMSF, 1.5 μM Pepstatin A,2.5 μM Leupeptin). Cell lysis was achieved through 3 sequential steps: first resuspended cells were exposed to one freeze thaw cycle, secondly lysozyme was added (0.1 mg/ml) for enzymatic lysis, and lastly cells were sheered through ultrasonification in presence of Triton-X-100 (0.4%), DNAse (5 μg/ml) and RNAse (10 μg/ml) (not in the case of the reporter of gene expression). Histidine- tagged proteins were purified using either Ni-NTA agarose and polypropylene columns, or His MagSepharose Ni2+ magnetic beads when only a small amount of protein was required. Proteins were eluated with elution buffer (20 mM Na_2_HPO_4_, 30 mM NaCl, 250 mM imidazole). Fluorescence spectra were recorded using either a Cary Eclipse Fluorescence Spectrometer (Varian), or an Infinite M200 PRO plate reader (Tecan).

### Cell culture, transfection and imaging

Sensors which were intended to be tested in HEK (293) cells, were subcloned into pcDNA3 (Invitrogen). Sensor DNA was delivered to HEK cells using calcium phosphate. For a dish with a volume of 2 ml, 1–5 μg of DNA were added to 87.6 μl H_2_0dd and 12.4 μl 2M CaCl_2_ prewarmed to 37°C. Subsequently, 100 μl of prewarmed BES buffer (50 mM BES, 280 mM NaCl, 1.5 mM Na_2_HPO_4_, pH 6.95) were added drop-wise to the mix, while it was constantly agitated. After 20 minutes of incubation at 37°C, the solution was added to the cells. After ~16 hours, the medium was changed and the cells were allowed to recover for two hours before they were ready for imaging.

HEK cells were imaged in glass bottom dishes coated with poly-L-lysine. The medium was replaced with HBSS (Hank’s Buffered Salt Solution) buffer (Gibco) containing 1 mM calcium and magnesium. The cells were imaged using an Axiovert 35M inverted fluorescent microscope (Zeiss). FRET changes in calcium sensors were induced with the addition of ionomycine to a final concentration of 0.6 μM.

## Results

### Imaging set-up and expression of sensors in bacteria

**Fig 1:**
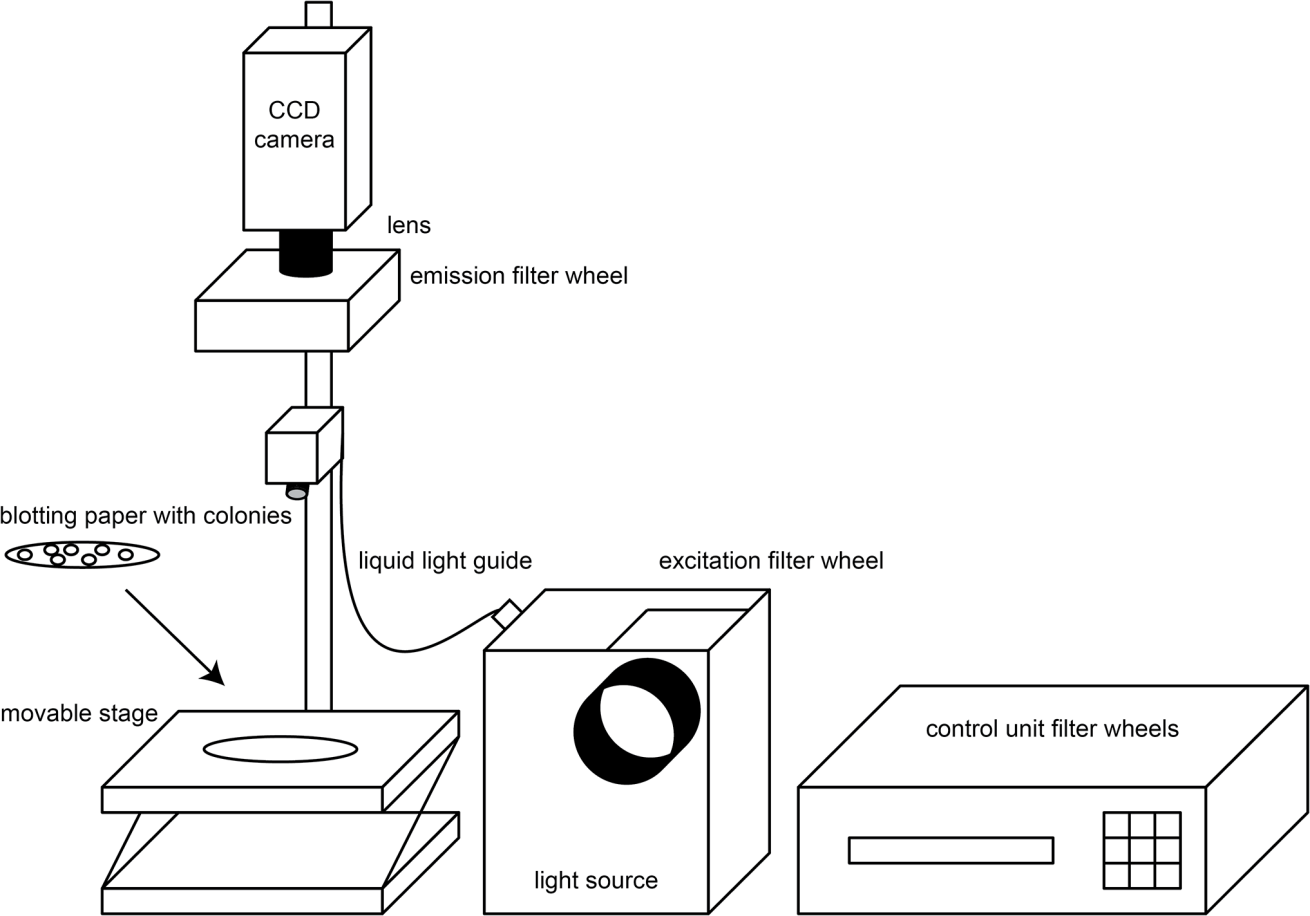
Scheme of the screening set-up. Wide-field CCD camera imaging of bacterial colonies blotted onto white paper is used for screening. The set-up consists of two filter wheels, a CCD camera, a lens and a movable stage for placing the colonies.

**Fig 2:**
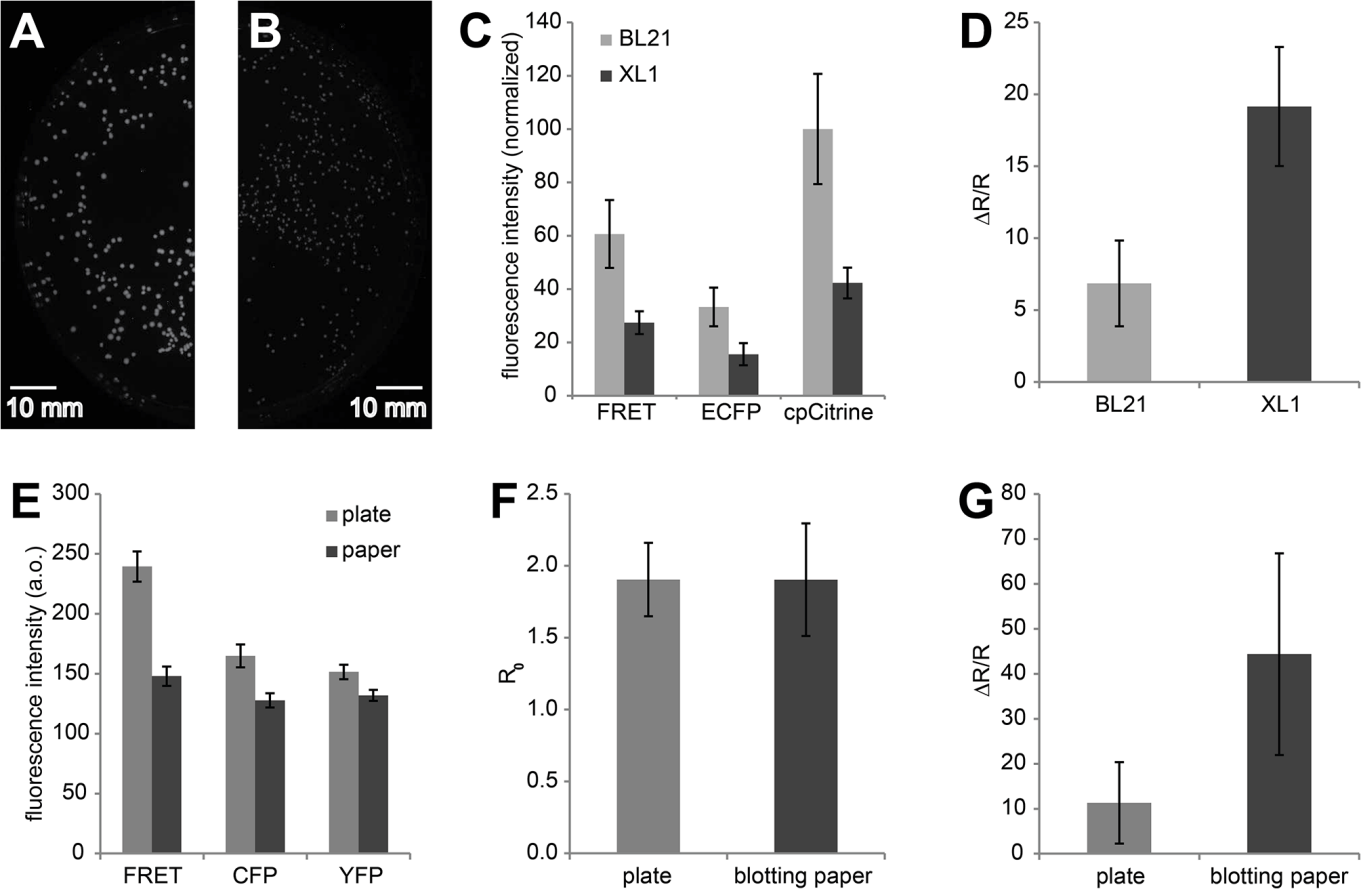
Optimization of expression conditions and screening background. **(A)** Colonies of *E*. *coli* BL21 transformed with TN-XXL cloned into the vector pRSETB (exposure time 4s, gain 2x, binning 1). Scale bar, 10 mm **(B)** Colonies of *E*. *coli* XL1 transformed with TN-XXL cloned into pRSETB and imaged under the same conditions. **(C)** Mean fluorescence intensities ± SD of colonies transformed with TN-XXL in either BL1 or XL1, imaged under identical conditions (n_BL21_ = 312, n_XL1_ = 558). **(D)** ΔR/R ± SD of *E*. *coli* colonies expressing TN-XXL cloned into BL21 or XL1 (n_BL21_ = 19, n_XL1_ = 15). **(E)** Auto-fluorescence of agar plate versus white blotting paper imaged under identical conditions with filters used for FRET imaging. Error bars indicate SD. **(F)** Basal ratio valuesR_0_ ± SD of XL1 colonies transformed with the FRET sensor TN-XXL and imaged on agar plate or on filter paper. (n_plate_ = 149 colonies, n_blotting paper_ = 90 colonies) **(G)** ΔR/R ± SD of the same colonies following calcium application.

The goal was to select sensors with large FRET changes out of a library of diversified sensors that would encompass a broad range of FRET ratio values both under resting conditions and in ligand bound state. These sensor libraries were transformed into bacteria and the bacteria were plated onto LB agar plates. We used a standard filter wheel set-up and wide-field imaging with a CCD camera to monitor the bacterial colonies expressing fluorescent biosensors (**Fig 1**). With this approach it could be ensured that a large number of sensor variants could be sampled simultaneously. Then we set out to optimize the bacterial strains used, expression and imaging conditions, permeabilization protocols to measure Rminin ligand free and Rmaxin ligand-bound state of the sensors, and software routines to automate colony identification and analysis of responses. We initially sought to find suitable conditions to express and image biosensors in *E coli*. For this purpose we used TN-XXL, a well described FRET-based calcium sensor [24,25], cloned into the bacterial expression plasmid pRSETB (Invitrogen), to test various bacterial strains and conditions. We compared sensor response in a bacterial strain used for protein expression, BL21-Gold (Stratagene), and a strain used for cloning, XL1-Blue (Stratagene) (**Fig 2**). As expected, BL21-Gold displayed a fluorescence intensity more than 2 times higher than that of XL1-Blue when transformed with vectors coding for TN-XXL (**Fig 2A–2C**).However, we typically observed that FRET changes, e.g. after permeabilization of the bacterial colonies for calcium, were consistently higher in XL1-Blue (**Fig 2D**). Lower, leaky expression of the biosensor in the strain XL1-Blue, which was intended not to express any recombinant proteins from transformed plasmids, avoided dense packing artefacts and other potential detrimental effects of protein overexpression in the bacterial cytosol. As the brightness in XL1-Blue still provided enough photons for screening we chose this strain for further work. Bacterial plates were transformed with biosensors and incubated at 37°C for about 16–18 hours. After that time, colonies were large enough to be used, yet no non-resistant satellite colonies were visible surrounding the main colonies. Subsequently, the plates were left at room temperature for another 5–6 hours to allow the proteins to further maturate, and then stored at 4°C overnight, before they were imaged on the second day. Colonies were blotted from agar plates onto blotting paper (Whatman 3MM) presoaked in MOPS buffer before imaging. This measure was thought to be beneficial since the agar plates exhibited autofluorescence which was reduced by white paper background (**Fig 2E**). Starting ratios (R_0_) of TN-XXL on plate and blotting paper were similar (**Fig 2F**). When treated with permeabilizing agents and high calcium however, the ΔR/R of TN-XXL was 4 times higher in colonies imaged on paper in comparison to those imaged on plates (**Fig 2G**). Presumably the blotting paper is more suitable to distribute the solution for permeabilization around the colonies to allow better penetration into the colonies. The blotting paper containing the colonies was then placed on a movable stage and imaged using the CCD camera set-up at room temperature (**Fig 1**).

### Permeabilization and imaging of bacterial colonies

**Fig 3:**
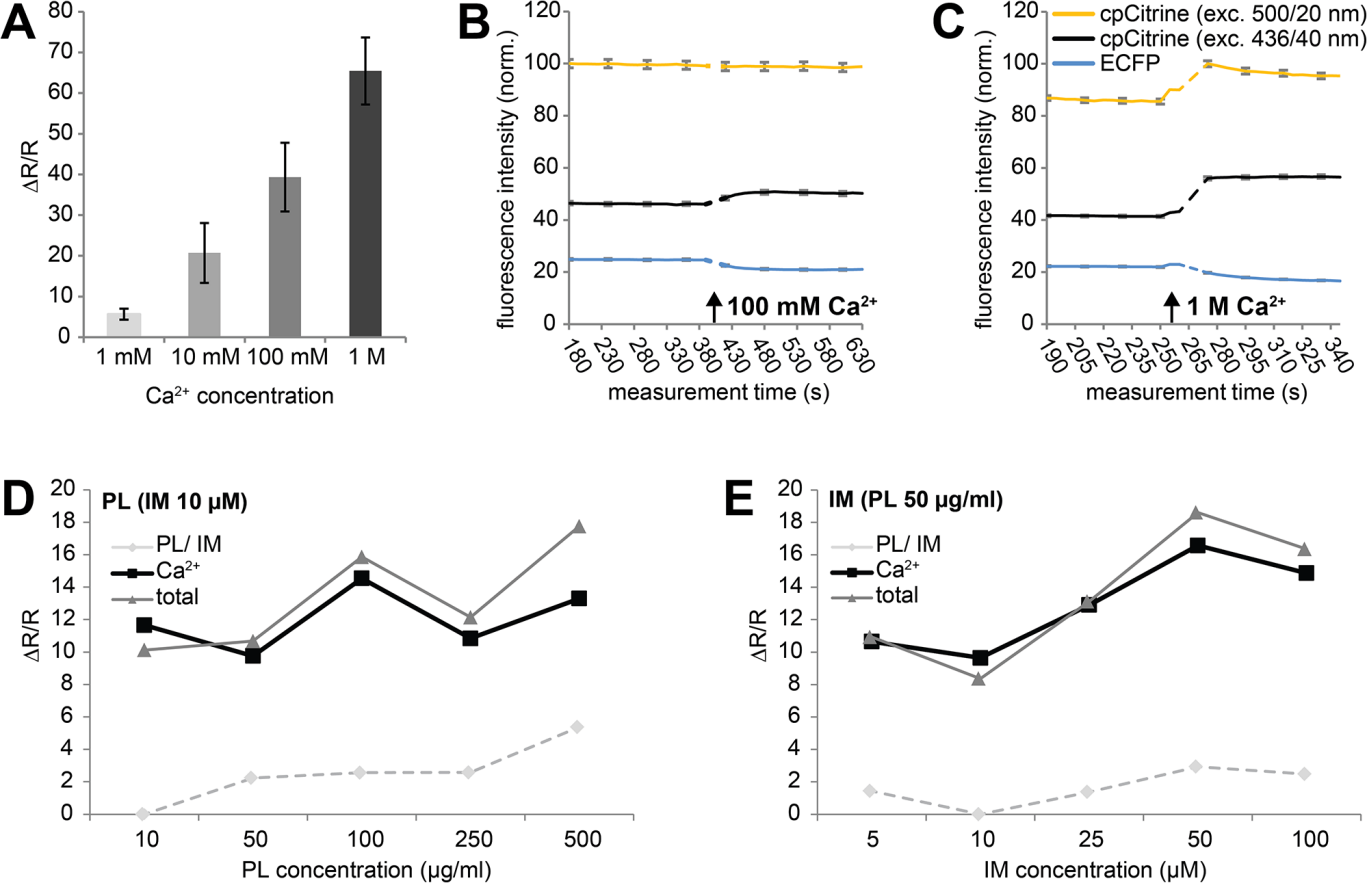
Permeabilizing *E*. *coli* to exogenous calcium **(A)** Average ΔR/R ± SD of a FRET sensor (Twitch-1) expressed in XL1 cells pretreated with poly-L-lysine and ionomycin and subjected to different concentrations of extracellular solution calcium (n = 6 colonies per concentration). **(B-C)** FRET trace (± standard error of the mean, grey) and individual donor and acceptor traces (± standard error of the mean, grey) of colonies of *E*. *coli* XL1 transformed with TN-XXL. Arrows indicate when calcium was applied. (n_100mM_ = 197 colonies, n_1M_ = 222 colonies). **(D-E)** Representative ΔR/R values following calcium application (100 mM) of colonies pretreated with different concentrations of either poly-L-lysine (PL) or ionomycin (IM), while the concentration of the second substance was kept constant. Grey dashed lines represent signal changes after the application of poly-L-lysine or ionomycin, black lines represent additional signal change after the application of calcium, and dark lines indicate total signal changes.

In order to measure Rmin and Rmax of a given sensor it is necessary to monitor the sensors in both ligand free and ligand bound conditions. Bacterial calcium under resting conditions is kept low in order to minimize calcium-induced toxicity [26, 27]. Thus, basal FRET measurements under these conditions provides a reasonable read-out of Rmin for calcium sensors. In order to obtain Rmax, it was necessary to open up the bacterial cell walls for calcium to penetrate and bind the sensor variants. Methylglyoxal, a carbohydrate metabolite that is thought to induce calcium transients in *E*.*coli* by opening calcium channels [28] was not found to be effective in our experimental set-up (data not shown). Thus, we decided to permeabilize the bacterial cell wall by other means. A number of chemicals were tested for that purpose to raise calcium and induce a detectable FRET change in bacterial strains of *E*. *coli* expressing TN-XXL. As a result of these attempts we identified a combination of the known calcium ionophore ionomycin [27] and the homopolymer poly-L-lysine [29] to be effective in reliably permeabilizing *E*. *coli* XL1-Blue and allowing measurement of Rmax in FRET calcium sensors. Different concentrations of the two compounds were tested, and the extracellular calcium concentration was increased to aid the action of ionomycin. The detectable ΔR/R of TN-XXL increased with higher calcium concentrations from 1 mM over 100 mM to 1 M (**Fig 3A**). However, when single wavelength recordings of experiments undertaken with 100 mM and 1 M calcium were compared, it became obvious that at 1 M calcium under direct acceptor excitation the intensity of the yellow fluorescent protein cpCitrine increased. Both Citrine [30] and cpCitrine have pKa values of 5.7. Most likely, high concentrations of extracellular calcium combined with ionomycin, which exchanges calcium against protons, alkalize the bacterial cytosol, thus increasing cpCitrine intensity independent of FRET. This undesired side effect was not observed with 100 mM calcium, (**Fig 3B and 3C**). Concentrations of either poly-L-lysine or ionomycin were optimized as well (**Fig 3D and 3E**). Thus, for screening calcium sensors, finally a mix of poly-L-lysine (50 μg/ml) and ionomycin (50 μg/ml) was applied, and subsequently the extracellular calcium concentration was raised to 100 mM. Solutions to permeabilize bacteria were initially applied by spraying four to five times (approximately 0.75 ml per blotting paper or plate) with a 30 ml commercial spray bottle (Rotert, Germany).

### Software and Data Processing

**Fig 4:**
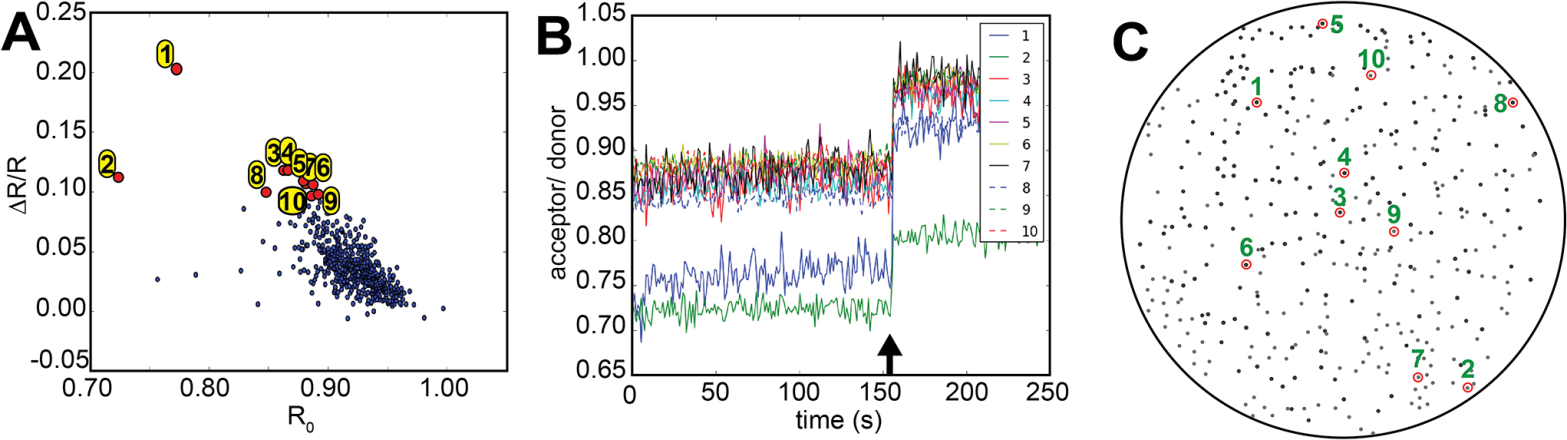
Imaging bacterial colonies expressing a FRET biosensor library. **(A)** Basal ratio R_0_ plotted against calcium-induced ratio change ΔR/R for every colony of one agar plate blotted onto white paper. Each colony is supposed to express a different diversified FRET calcium sensor variant. The 10 best performing colonies of this experiment are highlighted in yellow and numbered. **(B)** Single FRET traces of the 10 best colonies as identified in A. The arrow indicates when calcium was applied **(C)** Scheme of a screening plate with the positions of the 10 best performing colonies highlighted.

Analysis of FRET responses from a large number of bacterial colonies provided another challenge. We developed software in the programming language Python, used with the software package μManager [31] to control the camera, filter wheels and shutters and to guide the user through the screening process step by step, prompting certain actions at the appropriate time. Furthermore the program identified all colonies on a plate, recorded fluorescence intensities at all wavelengths of interest for those colonies, calculated FRET ratios, analyzed the data and identified the best performing colonies and sensors (**Fig 4**). In detail, fluorescent colonies were first imaged for YFP fluorescence, and the threshold for colony identification was set at 3σ of pixel intensity. This clearly found all the brightly fluorescent bacterial colonies on the non-fluorescent plate, generating a binary map of colony locations that was further processed with binary erosion and opening operations to clean up colony boundaries. Automated image segmentation was performed on the binary image, and the identified location and areas of the colonies were used to measure fluorescence intensities and calculate the FRET ratios for the colonies in real-time from subsequent images. The colonies were then ranked by ΔR/R_0_ divided by R_0_^0.5^, resulting in the selection of colonies according to their responses with a bias towards higher fluorescent changes rather than lower starting ratio. Following an experiment, the user was presented with three plots; a landscape of all sensors on the plate in respect to their R_0_ and ΔR/R, with a pre-selected number of well performing colonies in both criteria highlighted and labeled according to their ranking, single traces of those colonies, and lastly a scheme of the plate indicating the positions of the best colonies (**Fig 4A–4C**). This scheme could then be used to pick these colonies from the plate and retrieve the DNA coding for the expressed biosensor. The commented code was deposited at: https://github.com/GriesbeckLab/ColonySelection

### Analysis of variability

**Fig 5:**
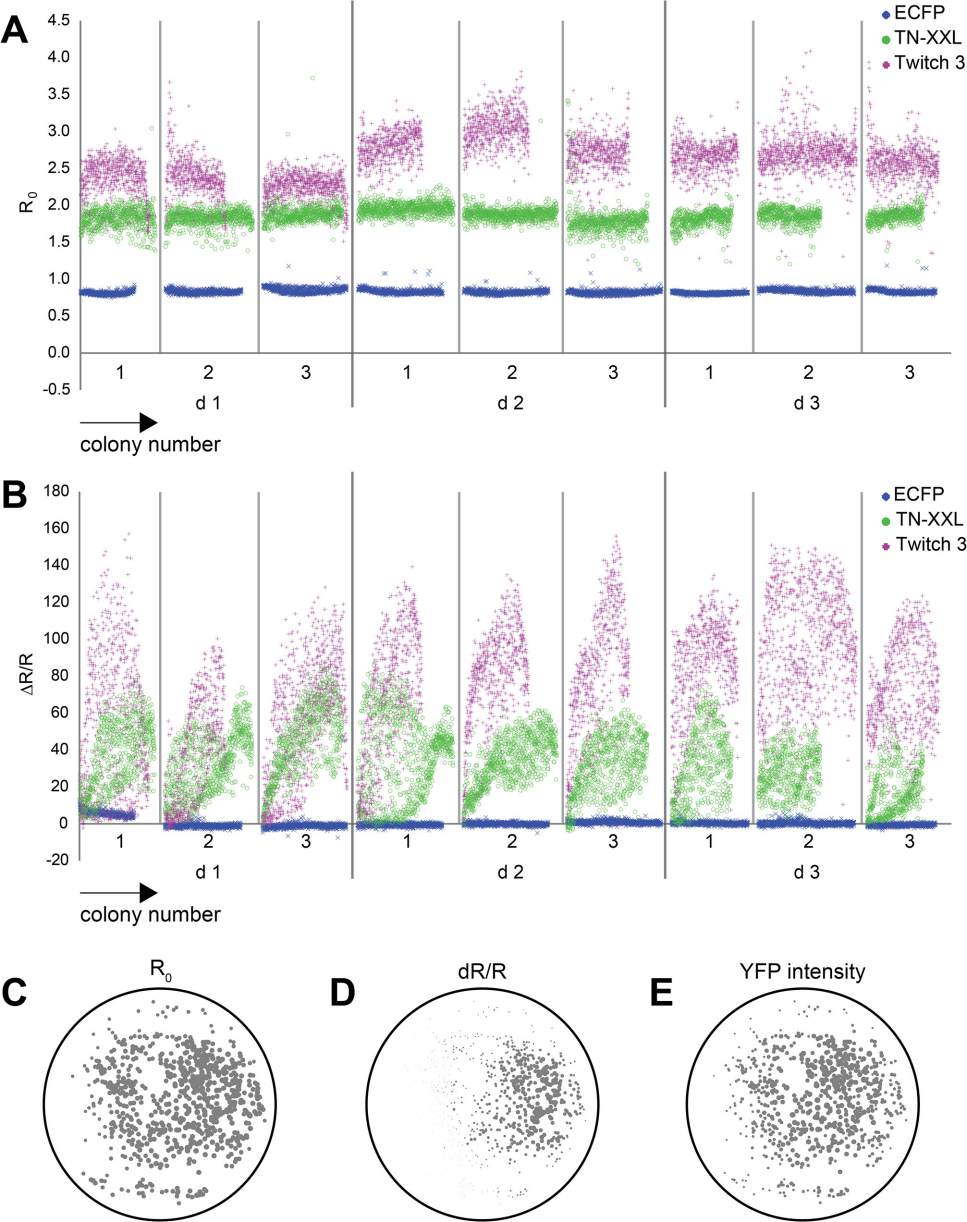
Analysis of variability. **(A)** Basal ratio R_0_ of all colonies on a plate expressing either ECFP or the FRET sensors TN-XXL and Twitch-3, ordered according to their position on the plate from bottom right to top left. 3 individual measurements were performed per day (1–3) on 3 different days after plating (d1-d3). **(B)** ΔR/R of the same colonies as shown in **(A)** after pretreatment with poly-L-lysine and ionomycin and application of calcium. **(C-E)** Bubble plot scheme showing dependency of a colony’s R_0_, ΔR/R and YFP intensity on the position on the blotted paper. Colonies express Twitch-3. Positions of colonies on a blotted paper are indicated by a circle at the corresponding position. The diameter of the circle indicates the relative value for R_0_, ΔR/R and YFP intensity. Note that basal ratio R_0_ is relatively independent of position, while the ΔR/R value shows a high degree of variability between colonies.

In order to test the reproducibility of the assay, the responses of one calcium-insensitive fluorophore and of two FRET sensors were compared. Our selection comprised the fluorescent protein ECFP, and the FRET sensors TN-XXL with an *in vitro* R_0_of ~0.95 and maximal ΔR/R of ~400% [24] and Twitch-3 with an *in vitro* R_0_ of ~1.30 and maximal ΔR/R of ~700% [32]. All three proteins were screened in separate experiments on three separate days, and variations in R_0_ and ΔR/R of each protein from experiment to experiment and from day to day were investigated. R_0_ values proved to be remarkably stable, and the three types of sampled proteins easy to distinguish from one another on the plates (**Fig 5A**). Rare outliers were mostly a result of the program wrongly identifying particles or the edge areas of blotting paper as regions of interest. ΔR/R values exhibited more variation from experiment to experiment especially on day 1 (where a considerable number of colonies expressing either TN-XXL or Twitch-3 could not be clearly distinguished from one another, whereas on day 3 this distinction was possible (**Fig 5B**). Index numbers were allocated to the colonies and position-dependent bubble plots were generated to correlate the position of a colony with its R_0_, ΔR/R or fluorescence intensity after direct cpCitrine excitation (**Fig 5C–5E**). Indeed, a clear correlation of position on the plate and ΔR/R was discovered, suggesting that all plates featuring large variations were not sprayed evenly by the experimenter (**Fig 5D**). The bubble plots of R_0_ and fluorescence intensity of cpCitrine under direct excitation displayed no dependence on position, confirming that the illumination of the plates was fairly even, with only a minor decrease at the edges of the plate (**Fig 5C–5E**).

**Fig 6:**
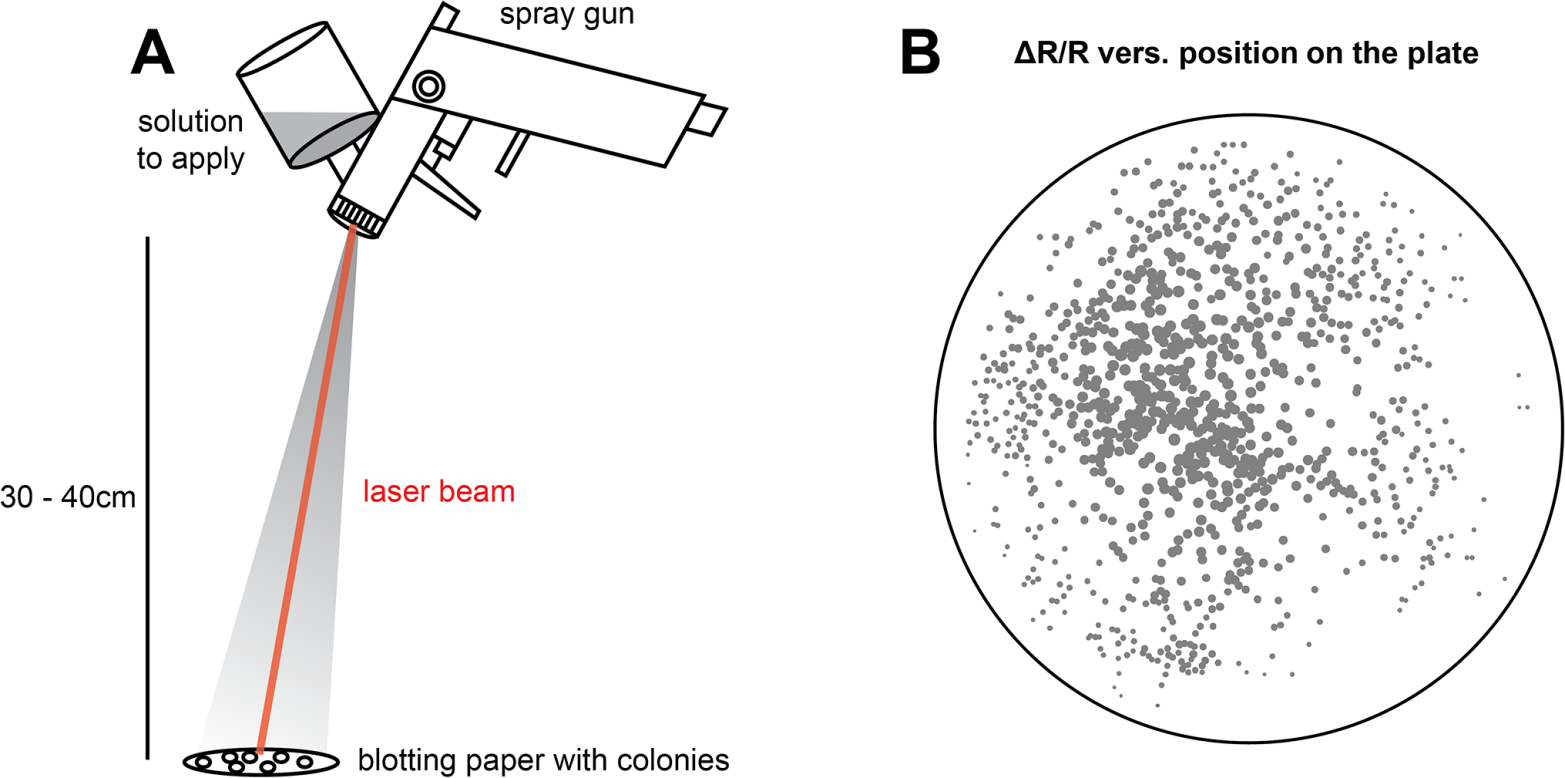
Improved spray devices allow more homogenous application of solutions. **(A)** A commercial spray gun is connected to a laser pointer to allow more reproducible aiming and application of solutions to bacterial colonies. **(B)** The improved spray protocol reduces variability in ΔR/R of bacterial colonies expressing the FRET calcium sensor Twitch-3. The position of each colony on a plate is indicated by a circle. The recorded ΔR/R value is represented by the diameter of the circle. Note that there is a gradient of ΔR/R values when moving from the center to the periphery.

In order to improve reproducibility, the way solutions were applied to the blotting paper was further improved. A spray gun (JetStream I, K350, HVLP), commonly used for spray painting, was utilized to spray on the solution. It permitted for a more even spraying pattern and offered a bigger spraying radius, allowing the experimenter to only aim for the middle of the plate instead of having to move the spraying device when trying to cover every part of the plate. In addition, a laser pointer was attached to the spray gun to allow reliable targeting (**Fig 6A**). The increased spraying radius reduced the error caused by the experimenter having to move the nozzle around whilst spraying, in an attempt to achieve full coverage. The build of the valve however, did account for a higher concentration of solution at the center, and a decrease towards the periphery. Analysis of signal change of a colony in respect to its position on the plate confirmed that signal changes were more reliable now (**Fig 6B**). A reduced ΔR/R was still visible towards the periphery of the blotting paper, but the differences were smaller. In detail, the coefficient of variation of the ratio change was 37% for the experiment conducted with the improved method using a spray gun (**Fig 6B**), compared to 67% for the experiment using a spray bottle (**Fig 5D**). As the decline of ΔR/R towards the periphery was very predictable, it may be taken into account when analyzing screening data.

### Sensor performance in bacterial colonies versus *in vitro*

**Fig 7:**
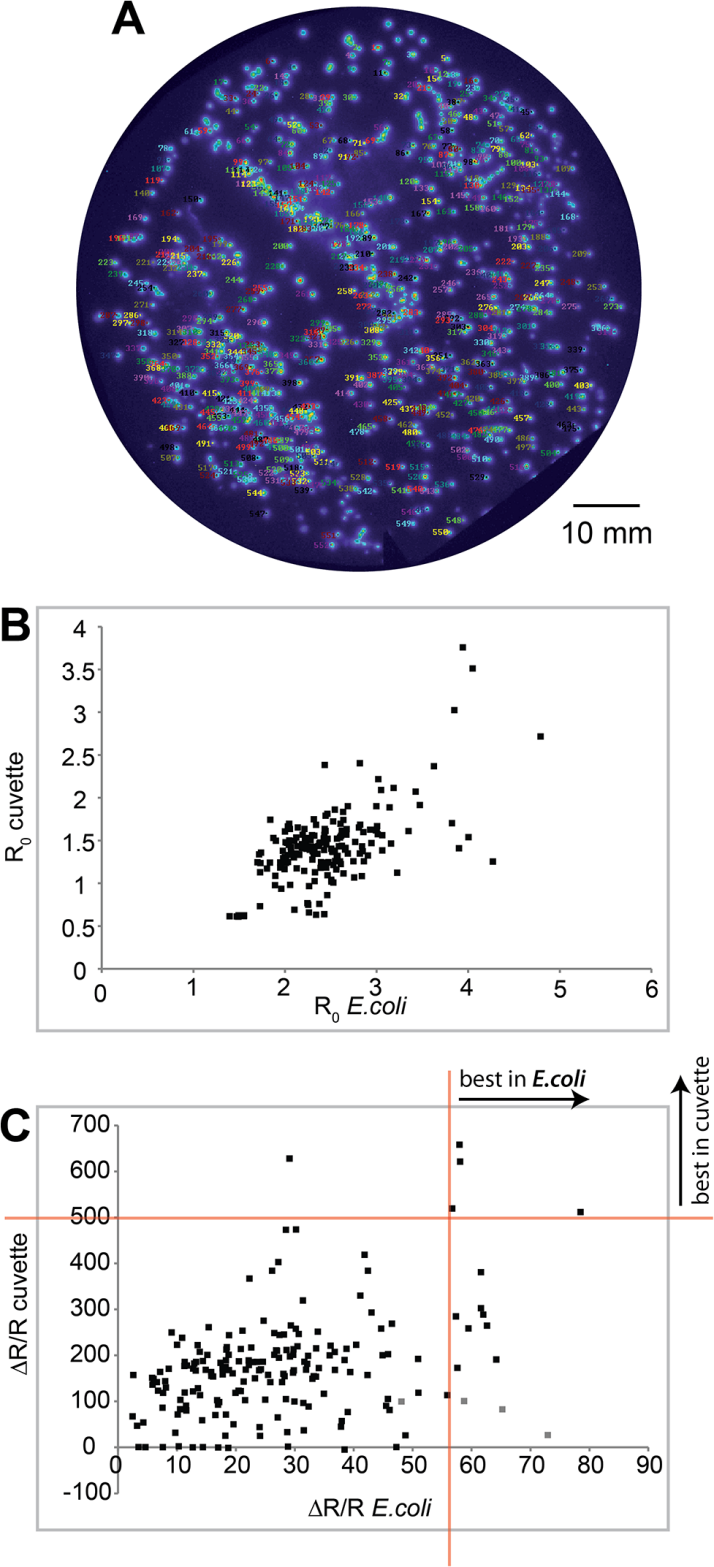
Sensor performance in bacterial colonies and *in vitro*. **(A)** Plate of *E*.*coli* colonies transformed with a library of diversified “Twitch” FRET sensors and blotted onto paper. Every colony was identified by software and automatically assigned an identification number. All colonies were imaged for calcium-induced FRET change, every 3^rd^ colony was picked, the corresponding sensor protein was purified and analyzed *in vitro*. **(B)** Comparison of starting ratios R_0_ measured in *E*.*coli* and in the cuvette. **(C)** Comparison of FRET ratio changes ΔR/R following calcium application in *E*.*coli* and in the cuvette. Red lines mark cut off criteria. The horizontal line is a cut-off marking the top 5 performing colonies as determined *in vitro*. The vertical line indicates the cut-off criteria for picking colonies after bacterial colony screening (approximately the best performing 2%). Thus, the colony screen was able to identify 4 out of the 5 best performing colonies *in vitro*.

To match data acquired from bacterial plates with *in vitro* data, an entire plate of colonies expressing a random library of sensors with diversified linkers was measured **(Fig 7A**). Libraries of FRET biosensors were generated as previously described [32,33].

In this particular case, a minimal calcium binding motif from the C-terminal lobe of Troponin C had been sandwiched between donor and acceptor fluorophores via randomized linker amino acid sequences between the calcium binding moiety and the fluorescent proteins. Linkers were created via PCR of the calcium binding domain with degenerate primers, allowing linkers of a length of 1–4 amino acids on each side of the minimal domain. Subsequent assembly of the sensor occurred using SLiCE (Seamless Ligation Cloning Extract) cloning [23]. Following bacterial colony screening by wide-field CCD camera imaging every third colony was picked, its sensor DNA was extracted and the sensor was expressed and purified to be tested *in vitro*. R_0_ and ΔR/R data from the bacterial plate was plotted against the data measured *in vitro* for every single sensor. R_0_ values acquired on bacterial plates and *in vitro* correlated well (**Fig 7B**), exhibiting a correlation coefficient (Pearson) of 0.61. ΔR/R values did not correlate as well (**Fig 7C**), with a correlation coefficient of 0.30 and some obvious mismatches. However, using the current protocol we have been able to identify four of the five best sensors, as determined *in vitro*, by only picking the best 15 colonies (approximately 2.5% of colonies on the plate) on the blotting paper, thereby reducing the number of variants to test considerably. Using this protocol we were able to enhance FRET ratio change in a minimal domain calcium sensor using CFP and cpCitrine as donor and acceptor fluorescent proteins from about 30% in the starting prototype to over 1000% in a sensor named Twitch-2 [32] by screening about 100.000 colonies (approximately 140 agar plates with bacterial colonies), demonstrating the practical usefulness of the procedure.

**Fig 8:**
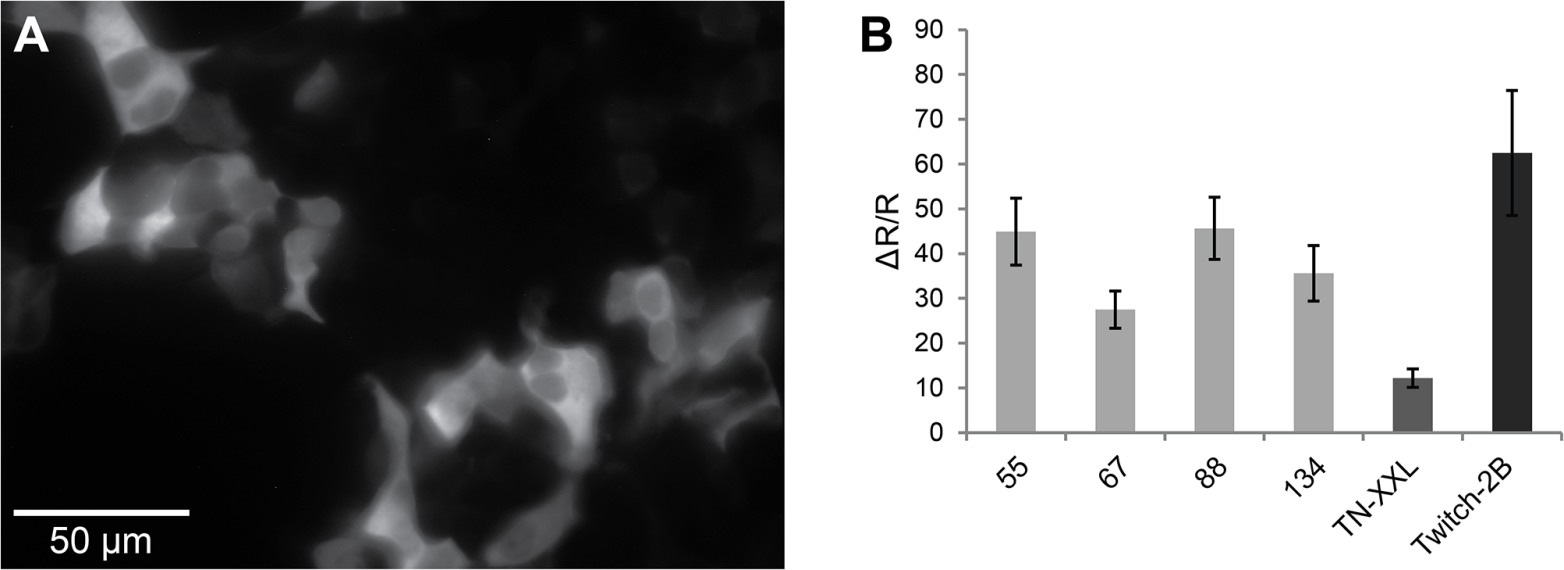
Sensors that do not perform after purification are functional in mammalian cells. **(A)** HEK cells expressing a diversified FRET calcium sensor variant (in this case number 55), YFP emission/CFP excitation. Scale bar, 50 μm. **(B)** Mean ΔR/R ± SD in HEK cells expressing a given variant and stimulated with ionomycin (0.6 μM) of the 4 sensor variants numbered 55, 67, 88 and 134, which are functional in *E*.*coli* but not *in vitro*, in comparison to the published sensors TN-XXL and Twitch-2B.

The data for **Fig 7** was obtained before the application of the permeabilization solution had been optimized, suggesting that inhomogeneous application of the solution had been one source for the low correlation. A number of sensors, which performed very well in *E*.*coli* but not well *in vitro* (marked in grey in **Fig 7C**) were expressed in HEK cells (**Fig 8**). Indeed, these sensors were fully functional and exhibited ratio changes comparable to some established sensors in cells. Thus, the purification procedure to obtain recombinant protein may have been detrimental for their function in cuvettes or alternatively, a cellular environment was needed for them to operate efficiently.

### Obtaining Rmax via inducible expression of a ligand

**Fig 9:**
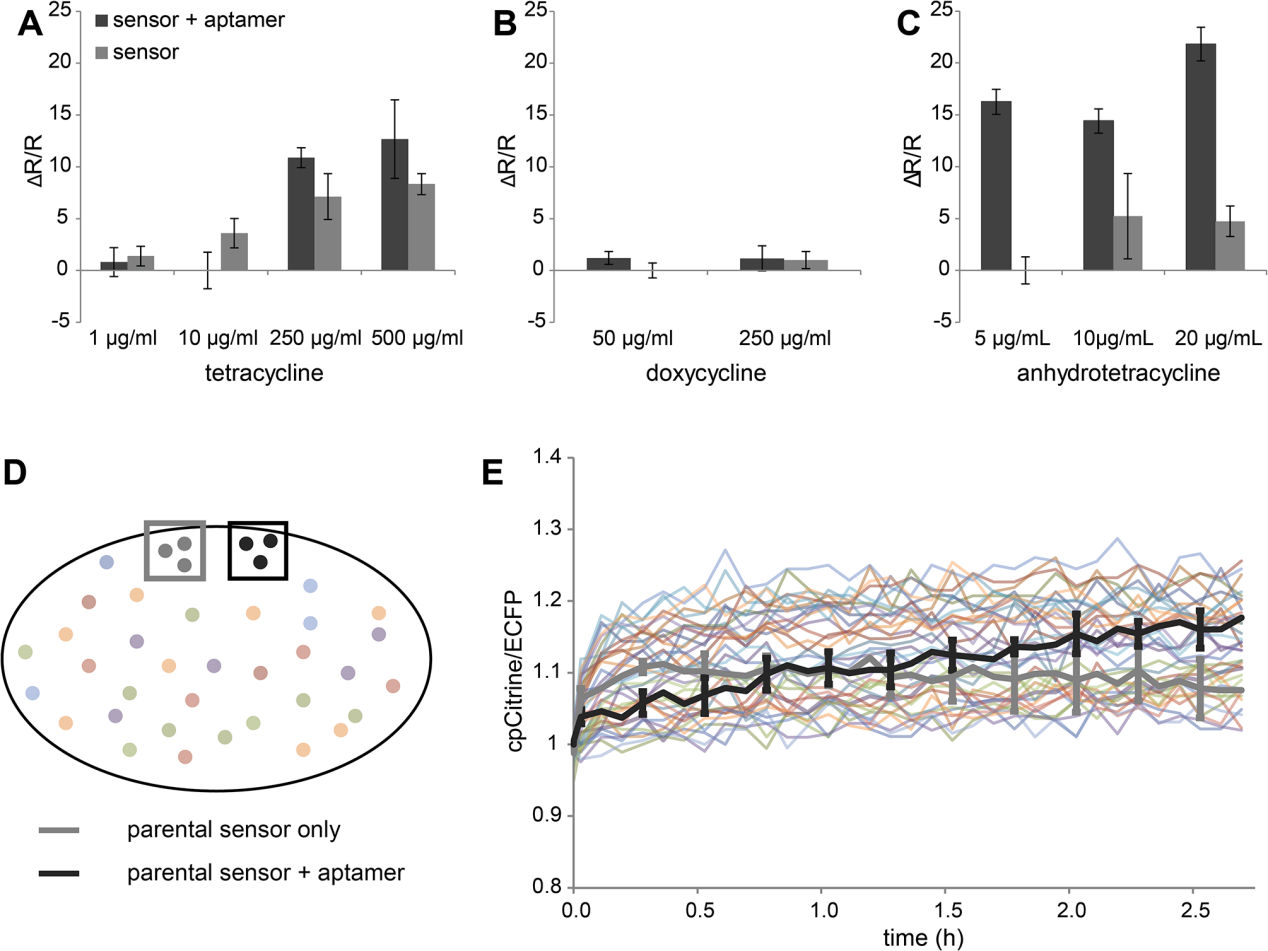
Optimizing small FRET changes with inducible ligands. A FRET reporter for gene expression was diversified and best sensor variants after induction of the RNA aptamer identified in bacterial colonies. **(A-C)** Mean ΔR/R values ± SD of cells transformed either with only the parental FRET sensor FR-Rsg1.2 [33], or a combination of the sensor with the corresponding RNA aptamer under the control of an inducible promoter following the application of tetracycline **(A)**, doxycycline (**B**) or anhydrotetracycline **(C)** (n = 4 for each drug). ΔR/R values were calculated from FRET basal ratios at the start of the experiment and after ~3 hours. **(D)** An agar plate of *E*.*coli* colonies expressing different versions of the sensor imaged together with the parental sensor FR-Rsg1.2 to be optimized (negative control) and the parental sensor co-transformed with the aptamer under the control of the inducible promoter (positive control). For this purpose pieces of agar plates with these reference colonies were excised and imaged along with each screening plate **(E)** FRET traces of single colonies expressing mutants of the sensor in comparison to the negative and positive control. Sensors which exhibited a higher ratio change compared to FR-Rsg1.2 after 4 hours were picked and the performance was analyzed *in vitro* (data not shown).

While calcium and other ligands need to be applied exogenously, there is an alternative way to obtain Rmax for certain FRET sensors for which a modifying enzyme activity could be induced in bacteria. Such enzyme could be a kinase, a protease or some other genetically encoded ligand, that, once induced, could work on the expressed sensor variant. As a proof of principle, we tested an approach to improve an RNA binding FRET reporter, consisting of the synthetic aptamer binding peptide Rsg1.2 sandwiched between 2 fluorescent proteins [33]. When the corresponding RRE (Rev Responsive Element) aptamer is expressed the Rsg1.2 peptide binds it and undergoes conformational change, thereby enhancing FRET. For this protocol of optimization we used a dual expression system, in which a copy of a FRET sensor variant is expressed on one plasmid, and a 10-fold concatemere of the RRE aptamer on a second plasmid under control of the inducible Tet promoter. *E*. *coli* XL1-Blue bacteria were co-transformed with the sensor library on one plasmid and the RRE aptamer on the other plasmid. The expression of the 10x RRE aptamer was induced via application of anhydrotetracycline (20ng/μl). An increase of ΔR/R was subsequently observed over the course of several hours. Tetracycline, and its closely related derivatives doxycycline and anhydrotetracycline were tested at different concentrations (**Fig 9**). In every experiment, bacterial colonies expressing the sensor alone served as a negative control in comparison to cells, which expressed the sensor in combination with the inducible aptamer. Tetracycline was tested at concentrations up to 500μg/ml. At 250 and 500 μg/ml, a higher ratio change could be observed in sensors which had been co-transformed with the inducible aptamer, indicating that the expression of the aptamer had been successfully induced. However, the difference between colonies containing the aptamer and those without was not significant (**Fig 9A**). With doxycycline, no expression of the aptamer could be observed (**Fig 9B**). Anhydrotetracycline on the other hand exhibited high efficiency in inducing aptamer expression even at low concentrations, and colonies expressing the aptamer could clearly be distinguished from those which did not (**Fig 9C**). Overall, FRET changes in this type of sensor were relatively small. Also note that tetracycline derivatives are fluorescent [34]. Addition of tetracycline in increasing amounts raised the apparent FRET ratio in controls when sprayed onto plates (**Fig 9A–9C**), reducing sensitivity of the assay. Thus, it was beneficial to screen the library in comparison to controls. A sample of colonies expressing either only the template FRET sensor (negative control), or the template FRET sensor together with the inducible aptamer (positive control), were imaged with the library for direct comparison (**Fig 9D**). Only colonies which exhibited a R_0_ value lower and a ΔR/R higher than the average of 6 colonies of the parental sensor expressed with the aptamer were picked and further analyzed (**Fig 9E**), demonstrating that it is feasible to optimize FRET sensors with relatively small changes and moderate improvements. Indeed, with this approach it was possible to enhance FRET change in VaMPIre, a reporter of gene expression and RNA localization, from initially 35% to approximately 240% [33].

## Discussion

Here we present procedures for bacterial colony based screening of relatively large numbers of diversified FRET sensors to optimize their FRET changes. These procedures are cost effective and allow rapid sampling of many variants of a given indicator. With suitable software the identification and picking of colonies expressing indicators with desired properties can be facilitated and the work load reduced considerably. Using these procedures we were able to dramatically increase the signal strength of two types of genetically encoded FRET indicators ([33,32]. It is foreseeable that with small differences in the compounds and protocol used to permeabilize the bacterial cell wall, other types of sensors can be screened as well. Potentially, bacteria could be permeabilized through other means [35].

One current major drawback of our method is uneven application of solutions for permeabilization of bacterial colonies, a problem that has been noticed previously [20]. This leads to variability of Rmax values in screening experiments which could result in missing a significant portion of interesting sensor variants. Use of commercial spray painting equipment lead to a more homogenous distribution of solutions, but more improvements here are certainly desirable. Replica plating of colonies has been proposed as an alternative to spraying solutions onto bacteria, but this poses a number of other challenges [20]. Nevertheless, the current assay is good enough to select candidate sensors with large FRET changes for further testing and characterization from an initially large pool. A secondary screen in a relevant cell type combined with a relevant physiological stimulus may be necessary for identifying sensors with appropriate affinity or response kinetics among the pre-screened pool. As example, we used the bacterial colony assay to pre-screen genetically encoded calcium sensors with large FRET changes [32]. The best performers were then fed into a secondary screen in primary hippocampal neurons [36] and the candidates with large responses to firing of small numbers of neuronal action potentials identified. In this way the number of indicators in an indicator screening and development pipeline can be narrowed down for use in more sophisticated high information content secondary screening that may be more costly and therefore only operating at low throughput.
